# Substance Use, Hospitalizations, and Co-Occurring Disorders among Patients Transferred from a Needle Exchange Program to Opioid Maintenance Treatment

**DOI:** 10.3390/ijerph19020697

**Published:** 2022-01-08

**Authors:** Martin Bråbäck, Anna Brantefors, Johan Franck, Louise Brådvik, Pernilla Isendahl, Suzan Nilsson, Katja Troberg, Anders Håkansson

**Affiliations:** 1 Department of Clinical Sciences Lund, Faculty of Medicine, Division of Psychiatry, Lund University, SE222 41 Lund, Sweden; Martin.Braback@med.lu.se (M.B.); Anna.Brantefors@skane.se (A.B.); louise.bradvik@med.lu.se (L.B.); Katja.Troberg@med.lu.se (K.T.); Anders_C.Hakansson@med.lu.se (A.H.); 2 Addiction Center Malmö, Division of Psychiatry, Lund University, SE222 41 Lund, Sweden; Suzan.Nilsson@skane.se; 3 Department of Clinical Neuroscience, Division of Psychiatry, Karolinska Institutet, SE171 77 Solna, Sweden; johan.franck@ki.se; 4 Department of Infectious Diseases, University Hospital Skåne, SE205 02 Malmö, Sweden; Pernilla.Isendahl@skane.se

**Keywords:** polysubstance use, psychiatric hospitalization, psychiatric co-occurring disorders, opioid maintenance treatment

## Abstract

Opioid use disorders (OUD) is a relapsing condition with high mortality. Opioid maintenance treatment (OMT) reduces heroin use, and overall morbidity and mortality. The prevalence of psychiatric and substance use disorders, potential baseline predictors for psychiatric hospitalization, and psychiatric diagnoses at follow-up were investigated and may give hints about possible preventative strategies. The medical records for 71 patients were reviewed 36 months following referral to OMT from a needle exchange program (NEP). Their psychiatric diagnoses and hospitalizations were identified. Their baseline characteristics were assessed for potential differences between hospitalized versus non-hospitalized patients and between patients with and without psychiatric diagnoses in a longitudinal observational study without controls. A regression analysis was performed to identify predictors for hospitalization when controlling for OMT status. Sixty-five percent of the patients were hospitalized at least once with a psychiatric diagnosis. Substance-related reasons were prevalent, and detoxification occurred among 59% of patients, with sedative- hypnotics (benzodiazepines, zopiclone, zolpidem, and pregabalin) being the substance used by 52% of patients. Baseline use of these drugs and/or buprenorphine predicted for hospitalization when controlling for OMT status. During the follow-up period, 72% of patients met the criteria for a psychiatric diagnosis other than OUD. The prevalence of non-substance use disorders overlapping with SUD was 41%, and that overlapping with anxiety disorder was 27% of all participants. Increased attention to psychiatric co-occurring disorders in the treatment of OUD is required and the importance of addressing sedative-hypnotics use when initiating OMT is highlighted.

## 1. Introduction

### 1.1. Opioid Use Disorders

Any opioid use disorder (OUD) puts patients at a lifelong risk for relapses and high mortality [1,2]. Opioid maintenance treatment (OMT) or Medication-Assisted Treatment (MAT), which includes psychiatric treatment and psychosocial intervention [3], is the most common treatment for opioid use disorders and has evidence with respect to reductions in drug use, criminal behavior, HIV risk behavior, and mortality [3,4,5,6,7]. OMT was introduced in the 1960s as a treatment for opioid use disorders [8], and at the turn of the century, buprenorphine was added as an alternative treatment option [4,9].

### 1.2. Co-Occurring Substance Use Disorders

Co-occurring substance use disorders are prevalent in opioid use disorders and include harmful use of alcohol, cannabis, stimulants, prescription opioids, or sedative-hypnotics (benzodiazepines, z-drugs (zopiclone and zolpidem), and pregabalin) [10,11,12,13]. When starting OMT, most individuals often report concomitant use of benzodiazepines [13,14]. The use of benzodiazepines during OMT has been associated with more psychopathology and a worse clinical course in several studies, with poly-drug use, risky injection practices, and poor psychosocial outcome [14,15,16,17,18,19]. Benzodiazepines may cause somnolence and confusion, contribute to mortality rates, and have been identified in up to 80% of methadone- or buprenorphine-related deaths [2,13,14,19,20]. Opioid users who also use benzodiazepines regularly are nearly three times as likely to have psychiatric hospitalizations during the preceding year [13].

### 1.3. Co-Occurring Psychiatric Disorders

Less data are available on the impact of co-occurring psychiatric disorders—other than substance-use related disorders—on the clinical course of opioid use disorders. The lifetime co-occurring disorders rates of other psychiatric disorders range between 44 and 86% [11,21,22]. Mood disorders and personality disorders are the most common, with the highest prevalence rates reported for major depressive disorder (4–44%) and antisocial personality disorder (ASPD) (25–39%), [12,23].

Psychiatric co-occurring disorders has a negative impact on drug dependence treatment outcomes in general [24,25,26,27].

Starting treatment with psychiatric disorders, such as major depression or antisocial personality disorder, is indicated to have a worse outcome [24,25]. However, for individuals in OMT, other studies suggest that psychiatric co-occurring disorders do not seem to have any significant impact on opioid use or retention in treatment [12,27,28,29,30]. Ngo et al. reported that, although comorbid heroin users entered treatment with a significantly higher risk of drug-related hospitalization than non-comorbid users, substantial reductions in drug-related hospitalization post-treatment were found [31]. The primary treatments for opiate dependence, such as methadone or buprenorphine maintenance or residential treatment, are associated with substantial improvements in depression [32].

The number of psychiatric beds in many high-income countries have been reported to have been reduced considerably since the late 1980s [33,34]. Some authors have therefore pointed out that being admitted to psychiatric in-patient care could be seen as a marker for a psychiatric condition with a high degree of severity [35].

### 1.4. The Present Cohort-Needle Exchange Program

We previously described the present cohort of individuals with OUD rapidly transferred from a needle exchange program (NEP) to OMT [36]. This low-threshold treatment aimed to increase accessibility, to avoid waitlists, to personalize treatment options, and to provide treatment design that focused on harm reduction, with a particular focus on the retention of low-adherence patients [37]. Despite reporting a high degree of psychosocial problems and poly-drug use, a majority of the 71 individuals that started OMT were still in treatment after 12 months [38]. The need for in-patient treatment after starting OMT and its relation to co-occurring disorders or baseline characteristics is, however, not thoroughly studied. A low-threshold OMT does not require abstinence and aims to reduce barriers to treatment. Therefore, other concomitant substance abuse may be expected as well as hospital admissions.

### 1.5. Purpose

The overall purpose of this study is to better outline the prevalence and manifestations of psychiatric co-occurring disorders in patients with opioid use disorders entering OMT after being referred from an NEP. The aims were to investigate the prevalence of psychiatric and substance use disorder-related hospitalization and potential baseline predictors of this among OMT patients with a 36-month follow-up from inclusion. Concurrent psychiatric diagnoses were also investigated. Therefore, possible preventative strategies could be proposed.

## 2. Materials and Methods

### 2.1. Study Design, Recruitment, and Inclusion/Exclusion Criteria

The study was performed in the city of Malmö located in the southern part of Sweden. The city has a population of approximately 300,000. The NEP is located in the university hospital area and run by the Department of Infectious Diseases. The outpatient OMT clinic is run by the Addiction Center Malmö. In Sweden, OMT is only permitted at special clinics supervised by a medical doctor and specialist in psychiatry or addiction medicine. The present study is a naturalistic follow-up of psychiatric co-occurring disorders and psychiatric hospitalization of patients receiving OMT.

The individuals were participants in the Malmö Treatment Referral and Intervention Study (MATRIS) [36] and were included between 21 October 2011 and 1 April 2013. Overall, 92% used heroin during the previous month. A treatment choice of either methadone or buprenorphine was made at the study inclusion and on an individual basis. The choice of medication and dosage was outside of the study protocol and based on individual clinical characteristics. The inclusion criteria were (1) primary injection use of heroin as documented in the NEP patient record, (2) having a minimum of two previous visits to the NEP office/treatment center, and (3) living in the region of Skåne, Sweden. Additionally, according to Swedish legislation, patients were required to be at least 20 years of age and to have had a diagnosed opioid use disorder for a minimum of one year [39]. Finally, for treatment initiation, a positive urine toxicology test for opioids (morphine derivatives, methadone, or buprenorphine) was required. The exclusion criteria were pregnancy, severe unstable psychiatric condition at baseline, other ongoing treatments for substance use disorders, and inability to understand information and to provide informed consent. Informed consent was given for 36-month follow-up from the date of inclusion. The study was approved by the Regional Ethics Review Board, Lund, Sweden, and registered at clinicaltrials.gov (No. NCT01457872).

In the primary MATRIS trial, 95% of included subjects were successfully referred to treatment [36], and the one-year retention among treatment initiators was 82% [38], similar to outcomes previously reported from a survey of OMTs in previous Swedish methadone maintenance treatment programs [40]. Out of the original 75 individuals who filled all of the criteria, 71 successfully entered treatment and constituted the analyzed cohort.

### 2.2. Baseline Data

**Figure 1:**
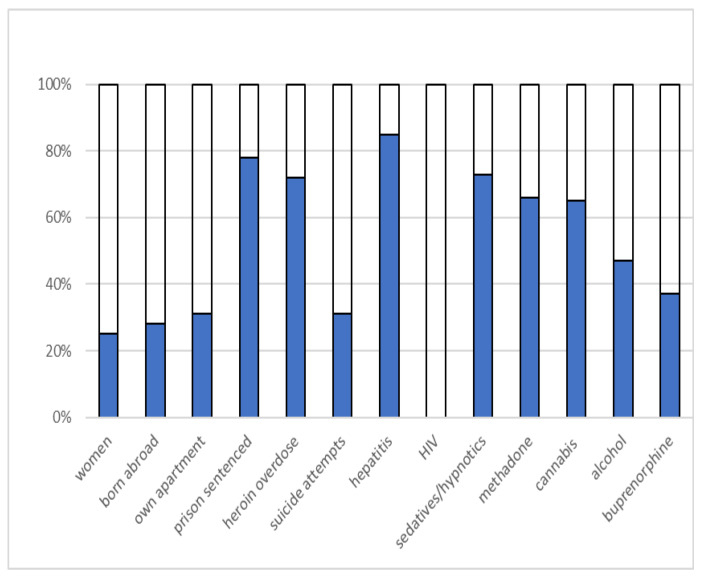
Sociodemographic factors.

The interview data included sociodemographic characteristics and self-reported data on substance-specific drug use and overdoses, previous suicide attempts, and history of psychiatric treatment (see Figure 1). A substance use assessment was adopted from the widely known Addiction Severity Index [41].

Opioid overdoses were defined as a lifetime history of opioid intake leading to difficulty in breathing, bluish skin, unconsciousness, or difficulty being woken up after intake of heroin. Drug use was specified for common illicit and prescription drugs with addictive potential, such as opioids, cannabis, central stimulating drugs, hallucinogenic drugs, and sedative-hypnotics. Based on their shared GABAergic effects and reports of a similar misuse pattern [42], benzodiazepines, z-drugs (zopiclone and zolpidem), and pregabalin were classified together as sedative-hypnotics. These drugs may cause somnolence and confusion and contribute to the risk of mortality [20,43]. Likewise, buprenorphine and buprenorphine-naloxone were grouped together. These drugs are increasingly used to self-medicate, often in combination with opioids, and are associated with self-harm, hospitalization, and opioid overdose mortality risk [44]. Detoxification from benzodiazepines was required when the medical risk in combination with OMT was high. Sometimes, out-patient treatment was adequate; sometimes, the patients applied for support from Social Security; and sometimes, in-patient detoxification was required.

Age, gender, housing, country of birth, previous suicide attempts, opiate overdose, and substance use at baseline were included as potential baseline predictors for psychiatric hospitalization at follow-up.

### 2.3. Outcome Measures

Patient records were reviewed in the medical database (software Melior, Siemens AB, Upplands Väsby, Sweden) from the date of inclusion in MATRIS and three years after. The focus of the reviews were psychiatric co-occurring disorders and treatment in two aspects: (1) hospitalization and (2) psychiatric diagnosis in their medical record, both measured over 36 months of follow-up.

Reasons for hospitalization were defined both by the diagnoses [45] and measures taken at the hospital stay from the text of medical records. All psychiatric diagnoses (ICD-10 codes F1-F9) from the case records were included. The number of admissions as well as the duration of time spent in psychiatric in-patient care were recorded.

### 2.4. Statistical Analysis

Baseline characteristics were assessed for potential differences between hospitalized versus non-hospitalized patients and between patients with and without a psychiatric diagnosis. Comparisons were made using a Chi-square test for binary variables and Fisher’s exact test whenever less than five observations were found in one category. For age, the Mann–Whitney test was used. The level of significance was set at *p* < 0.05. Finally, a logistic regression analysis (95% CI) was used to adjust potential baseline predictors for one another.

A Cox regression proportional hazard analysis with time-varying co-variates was performed to determine whether discharge from OMT predicted hospitalization while controlling for other variables that were significantly associated with hospitalization.

Both the logistic regression and the Cox regression proportional hazard analysis were carried out as a direct, non-stepwise analysis involving variables that were significantly associated with the outcome in the non-adjusted, bivariate analyses.

SPSS Statistics, version 22 (IBM Corp., Armonk, NY, USA), was used to perform the statistical analysis.

## 3. Results

### 3.1. Psychiatric and Substance Use Disorders Related to Hospitalization

During 36 months of follow-up, 65% were hospitalized on at least one occasion with a psychiatric diagnosis. Descriptive information on the reasons for hospitalization and its prevalence are presented in Table 1. The results show that substance-related reasons were the most prevalent and that detoxification occurred for 59% of the study population.

Detoxification from sedative-hypnotics was required by more than half (52%) of the study participants, all of which were for benzodiazepines and z-drugs (zopiclone and zolpidem) and some of which were for additional pregabalin.

### 3.2. Potential Baseline Predictors of Hospitalization

Use of addictive non-opioid drugs (by self-report) at baseline apart from opioids were common, mainly sedative-hypnotics (73%), alcohol (47%), amphetamine (32.4), and cannabis (19.7%).

Baseline use of sedative-hypnotics, and buprenorphine were both found to be significantly associated with hospitalization (Table 2). When controlling these two variables for one another in a logistic regression, hospitalization was only significantly associated with baseline use of buprenorphine (OR 4.2, CI 1.2–14.5, *p* = 0.025) and not with baseline use of sedative-hypnotics (OR 3.1, CI 1.0–9.8, *p* = 0.052).

When performing a Cox proportional hazard regression with time to discharge as a time-varying covariate, hospitalization was significantly predicted by discharge from OMT (HR 2.0, CI 1.0–3.9, *p* = 0.039). Likewise, hospitalization was predicted by baseline use of sedative-hypnotics (HR 2.6, CI 1.2–5.7, *p* = 0.015), or buprenorphine (HR 2.3, CI 1.3–4.3, *p* = 0.005).

### 3.3. Comorbid Psychiatric Diagnoses

Psychiatric co-occurring disorders was found to be highly prevalent. Descriptive information on psychiatric diagnoses within three years from inclusion and their prevalence are provided in Appendix A. In the three-year period, 72% of the study participants obtained a psychiatric diagnosis other than opioid dependence. Overall, 63% had some kind of SUD and 32% of the sample were dependent on sedative-hypnotics. When excluding SUDs, less than half of the individuals (41%) were diagnosed; the most prevalent subgroup was anxiety disorders (27% of all participants).

No baseline characteristic was found to predict a non-SUD psychiatric diagnosis (Table 3).

## 4. Discussion

The results of this study showed that most patients with OUD who had been referred from an NEP to OMT needed psychiatric hospital care during the 36-month follow-up, mainly for substance-related reasons, among which sedative-hypnotics detoxification predominated. Baseline buprenorphine predicted a significant risk of hospitalization. Both buprenorphine, and sedative-hypnotics predicted time to first hospitalization. Psychiatric co-occurring disorders was common, mainly SUD, and among non-SUD patients, anxiety was the most common.

One of the main findings was the predominance of detoxifications from sedative-hypnotics (benzodiazepines and z-drugs (zopiclone and zolpidem)), with 52% of in-patient treatment episodes in this group of patients with primary opioid dependence. Studies that report the rates of detoxification are sparse, to the best of our knowledge. One study showed that 18% of the patients in traditional OMT were admitted for detoxification at least once [15]. However, that study had a shorter follow-up, at most 24 months, and an unknown proportion of patients. Therefore, that study is not quite comparable with this one. One could expect the need for in-patient detoxification to be higher in patients referred from an NEP to OMT due to a high degree of polysubstance use at baseline. Preventative measures are important.

Previous research has shown that patients in treatment for opioid dependence—typically opioid use disorders—often misuse benzodiazepines either for the purpose of self-medication of psychiatric symptoms, for the purpose of becoming ‘high’, or in order to potentiate the effects of the prescribed opioid maintenance medication or an illicit opioid including heroin [44,46,47]. This has been explained as a ‘heroin-like’ effect of benzodiazepines when taken with methadone as well as enabling a prolonged effect of the illicit drugs and a smoother withdrawal [13].

Sedative-hypnotics (benzodiazepines, z-drugs (zopiclone and zolpidem), and pregabalin) contribute to opioid overdose mortality [13,19,48,49,50] and are present in 10–80% of methadone-related deaths and in 80% of buprenorphine-related deaths [13,14]. Concomitant use of benzodiazepines, including prescribed doses, increases sedation and interferes with cognitive function in patients maintained on methadone or buprenorphine and affects physical parameters when taken together with methadone [14]. Furthermore, the misuse of benzodiazepines during OMT is associated with continued use of opioids and illicit drugs, and problems with mental health [17,18,51,52]. Given the benzodiazepine-related harms in opioid-dependent patients, researchers have called for increased focus on the treatment of psychiatric co-occurring disorders in this condition to decrease the reasons for self-medication use of benzodiazepines [44]. Finally, dispensation of sedative-hypnotics, or benzodiazepines to patients in OMT are common and have pros and cons [53]. It has recently been shown that a substantial minority of OMT patients in Sweden were dispensed benzodiazepines (15%) and z-hypnotics (26%) [54]. Other sources of prescription are of course also possible. (Patients at the present unit were never prescribed these drugs.)

Non-prescribed use of buprenorphine prior to treatment start was a significant predictor of hospitalization and remained so after controlling for baseline use of sedative-hypnotics. Whereas this finding is difficult to interpret, it can be hypothesized that patients were using this as a treatment rather than recreationally, as supported by the literature [55]. A recent review also reported results indicating that most people using illicit buprenorphine did so for reasons related to misuse, that is, as efforts to manage opioid withdrawal symptoms or to achieve or maintain abstinence rather than a motive to get high [56]. Intuitively, this non-prescribed use of buprenorphine might not necessarily be associated with a more severe course in treatment.

This result suggests that sedative-hypnotic drugs constitute a significant clinical problem, as shown by the prediction of hospitalization, and—despite not being the patient’s primary drug of abuse—as the most common reason for it during OMT.

Psychiatric co-occurring disorders were common, and SUD predominated. The proportion of patients that received a non-SUD psychiatric diagnosis in this study was 41%. Studies in similar patient populations have reported 44–86%, albeit for lifetime prevalence [11,12,21,57]. However, in a large study of co-occurring disorders among OMT patients using SCID (Structured Clinical Interview for DSM-IV-Axis I Disorders) at 1–8 weeks, as many as 75% had a non-SUD psychiatric disorder [12]. When excluding other substance use disorders, the prevalence of psychiatric disorders in the present study was comparable with earlier reports [11,21,22,48], although lower rates have been reported from China, where only 30% matched criteria for a non-SUD psychiatric [57]. The most common non-SUD diagnosis was anxiety, with 27% of all participants. As mentioned above, researchers’ have called for increased focus on the treatment of co-occurring disorders to decrease the reason for self-medication with benzodiazepines [44]. Anxiety and misuse could be assumed to be correlated. Persons with anxiety may start self-medication, and benzodiazepines could sometimes worsen anxiety. Both problems should be identified and addressed.

Standing out in the literature is the high prevalence of personality disorders, with estimations of 25–40% having antisocial personality disorder (ASPD) [12,57]. Our finding that only 7% fulfilled a personality disorder diagnosis and one single individual had ASPD is probably an underestimation of the actual prevalence. This is probably since we used ICD diagnoses at in-patient or out-patient admissions, where personality diagnoses had lower priority and could not be easily evaluated due to drug use, lack of diagnostic instruments, etc.

The present findings on SUD show a clinically relevant prevalence, particularly as 32% were diagnosed with sedative-hypnotics dependence and 31% diagnosed with poly-substance drug use dependence (notably, these overlap to a large extent). However, this may be compared with past-month misuse estimates ranging from 7–73%, with a majority (67%) of studies reviewed reporting rates greater than 40% for people in treatment for OUD [58]. That is in the upper end and could be expected, as compared with studies that mostly had high threshold admittance. The rate of self-reported misuse at baseline was 73%, which may also be compared with the same estimates.

Recent studies have suggested that the use of benzodiazepines is negatively associated with retention in OMT [59,60,61], whereas other studies failed to demonstrate such an association [16,17].

The importance of socio-demographics for treatment outcome and psychiatric severity including gender differences in co-occurring disorders has been reported [62]. Associations between SUD and specific psychiatric disorders (including other SUDs) have been investigated as underlying factors of co-occurring disorders [10]. The results showed weak but significant associations [10].

The number of psychiatric beds in many high-income countries have been reported to have been reduced considerably since the late 1980s [33,34]. Some authors have therefore pointed out that being admitted to psychiatric in-patient care could be seen as a marker for a psychiatric condition with a high degree of severity [35]. Therefore, the present study could be presumed to deal with high severity, and some less serious cases may have been missed out.

An important limitation of the present study is the final sample size, as it reduces the number of co-variates by as much as possible to include potential baseline predictors of outcome. The small sample size may affect interpretation and generalizability. Information about the clients who chose not to participate in the present study is limited.

No structured diagnostic assessment was made, and therefore, the diagnostic accuracy cannot be regarded as optimal. However, the study design with one single site and diagnostic interviews mainly performed by the same psychiatrist should reduce diagnostic random errors and treatment confounders to some degree. Regardless, as secondary diagnoses were also included—with the purpose of identifying co-occurring disorders—another issue is that diagnoses tend to persist and that diagnoses set before OMT initiation may routinely but wrongfully have been repeated during hospitalization. Although a structured diagnostic instrument such as the (SCID) [63] would have improved specificity, its validity would have been hampered by patients’ current drug use.

Finally, as the present study deals with NEP, the findings may not be sufficiently applicable to general OMT. Populations attending SEPs have been previously reported to have a high degree of drug use severity and a high prevalence of psychiatric co-occurring disorders compared with other opioid users [64]. When referred to maintenance treatment, SEP attendees have also been reported to have a poorer treatment response [65].

## 5. Conclusions

In conclusion, most patients who entered standard OMT following referral from a needle exchange program (NEP) with psychological and psychosocial treatment needed psychiatric hospital care. Baseline drug use of sedative-hypnotics, and buprenorphine predicted a worse clinical course with respect to psychiatric in-patient care for three years following OMT initiation. The problem of buprenorphine use needs further exploration. This study supports the assumption that psychiatric diagnoses other than SUD are common among patients enrolled with OUD, with anxiety disorders being the most prevalent co-occurring disorders. This requires increased attention toward psychiatric co-occurring disorders in the treatment of OUD and highlights the importance of addressing sedative-hypnotics use when initiating OMT.

## Figures and Tables

**Table 1:** Participant prevalence of psychiatric and substance use disorders and other reasons for hospitalization. (N = 71).

Reason	*n* (%)
**Suicide prevention**	7 (9.9)
**Psychosis**	2 (2.8)
**Substitution treatment adjustments**	10 (14.1)
**Intoxication**	7 (9.9)
**Detoxification, all**	42 (59.2)
**Any from opioids**	21 (29.6)
**Any from benzodiazepines**	37 (52.1)
**Any from alcohol**	5 (7.0)
**Any from amphetamine**	9 (12.7)
**Any from cannabis**	16 (22.5)
**Any hospitalization**	46 (64.8)

**Table 2:** Participant characteristics of subjects with and without psychiatric hospitalization by self-report at baseline. (N = 71).

Demographics, Self-harm, and Addiction	Hospitalized, *n* (%) 46 (64.8)	Non-Hospitalized, *n* (%) 25 (35.2)	*p*
Age (Median)	37.4	34.6	0.342
**Gender**			0.847
Women	12 (26.1)	6 (24.0)	
Men	34 (73.9)	19 (76.0)	
**Country of birth**			0.981
Nordic	33 (71.7)	18 (72.0)	
Non-Nordic	13 (28.3)	7 (28.0)	
**Housing**			0.981
Permanent	33 (71.7)	18 (72.0)	
Non-permanent	13 (28.3)	7 (28.3)	
**Previous self-harm** Suicide attempt	17 (37.0)	5 (20.0)	0.140
Overdose opiates	35 (76.1)	16 (64.0)	0.280
**Baseline use** Sedative-hypnotics ^(1)^	38 (82.6)	14 (56.0)	0.016 *
Alcohol	23 (50.0)	10 (40.0)	0.420
Buprenorphine	22 (47.8)	4 (16.0)	0.010 **
Methadone	28 (60.9)	19 (76.0)	0.198
Tramadol	10 (21.7)	2 (8.0)	0.193
Fentanyl ^(2)^	2 (4.2)	1 (4.0)	1.0
Other opioids ^(3)^	17 (37)	6 (24)	0.265
Amphetamine	17 (36.9)	6 (24.0)	0.265
Cocaine	6 (13.0)	4 (16.0)	0.733
Cannabis	30 (65.2)	16 (64.0)	0.918
LSD	0 (0.0)	0 (0.0)	-
Ecstasy	1 (2.2)	0 (0.0)	1.0
Methylphenidate ^(2)^	3 (6.5)	0 (0.0)	0.547

* *p* < 0.05 Chi-square test. ** *p* < 0.05 Fisher’s exact test. ^(1)^ all used benzodiazepines/z-drugs(zopiclone, zolpidem), some pregabalin in addition. ^(2)^ 17 participants were not asked about Methylphenidate and Fentanyl. ^(3)^ including pain killers, such as oxycodone, codeine, and morphine. Chi-square test was performed for binary variables. When there were *n* < 5 observations in one or more categorical variables, Fisher’s exact test was performed. For continuous variables, i.e., age, Mann-Whitney test was performed. The level of significance was set to *p* < 0.05.

**Table 3:** Participant characteristics of subjects with and without psychiatric diagnosis *.

Variables	Psychiatric Diagnosis * *n* (%) 29 (40.8)	No Psychiatric Diagnosis * *n* (%) 42 (59.2)	*p*
**Age (Median)**	33.5	38.2	0.167
**Gender**			0.845
** Women**	7 (24.1)	11 (26.2)	
** Men**	22 (75.9)	31 (73.8)	
**Country**			0.129
** Nordic**	18 (62.1)	33 (78.6)	
** Non-Nordic**	11 (37.9)	9 (21.4)	
**Housing**			0.656
** Permanent**	20 (69.0)	31 (73.8)	
** Non-permanent**	9 (31.0)	11 (26.2)	
**Previous** **Suicide attempt**	11 (37.9)	11 (26.2)	0.293
** Overdose opiates**	23 (79.3)	28 (66.7)	0.244
**Baseline use** **Benzodiazepine/pregabaline**	23 (79.3)	29 (69.0)	0.337
**Alcohol**	15 (51.7)	18 (42.9)	0.462
**Suboxone/Subutex**	11 (37.9)	15 (35.7)	0.849
**Methadone**	20 (69.0)	27 (64.3)	0.682
**Tramadol**	6 (20.7)	6 (14.3)	0.479
**Fentanyl ^(1)^**	2 (6.9)	1 (2.4)	0.563
**Other opiates ^(2)^**	12 (41.2)	11 (26.2)	0.179
**Amphetamine**	9 (31.0)	14 (33.3)	0.839
**Cocaine**	6 (20.7)	4 (9.5)	0.298
**Cannabis**	19 (65.5)	27 (64.3)	0.915
**LSD**	0 (0.0)	0 (0.0)	-
**Ecstasy**	1 (3.4)	0 (0.0)	1.000
**Methylphenidate ^(1)a^**	2 (6.9)	1 (2.4)	0.563

* Other than substance use disorder. ^(1)^ 17 participants were not asked on Ritalin/Concerta and Fentanyl. ^(2)^ including pain killers, such as oxycodone, codeine, and morphine. Chi-square test was performed for binary variables. When there were *n* < 5 observations in one or more categorical variables, Fisher’s exact test was performed. For continuous variables, i.e., age, Mann-Whitney test was performed. The level of significance was set to *p* < 0.05. Benzodiazepine and pregabaline are grouped together, as they are both sedative-hypnotics. Suboxone and Subutex are grouped together, as they are both buprenorphine. ^a^ This was the only item, which not all of the participants responded to.

## Data Availability

The data presented in this study are available on request from the corresponding author. The data are not publicly available due to data protection regulations by the ethics board and by the regional hospital authority, and can be shared in anonymized format only after review by these authorities.

## Appendix A

**Table A1:** Information on psychiatric diagnoses within three years from inclusion and their prevalence.

	*N* (%)
Substance Use Disorder (SUD) * (F1) **	45 (63.4)
Alcohol	
Abuse	4 (5.6)
Dependence	6 (8.5)
Cannabis	
Abuse	3 (4.2)
Dependence	6 (8.5)
Sedatives and hypnotics	
Abuse	3 (4.2)
Dependence	23 (32.4)
Cocaine	
Abuse	1 (1.4)
Dependence	0 (0.0)
Stimulants	
Abuse	2 (2.8)
Dependence	5 (7.0)
Volatile solvents	
Abuse	0 (0.0)
Dependence	1 (1.4)
Poly-substance drug use	
Abuse	1 (1.4)
Dependence	22 (31.0)
**Psychotic disorders (F2)**	**2 (2.8)**
Non-organic psychotic disorder	2 (2.8)
Non-organic psychosis	1 (1.4)
**Mood disorders (F3)**	**8 (11.3)**
Bipolar disease	1 (1.4)
Depression	6 (8.5)
Unspecified	1 (1.4)
**Anxiety disorders (F4)**	**19 (26.8)**
Generalized Anxiety Disorder (GAD)	1 (1.4)
Mixed anxiety/depressive disorder	12 (16.9)
Anxiety, unspecified	8 (11.3)
**Personality disorders (F6)**	**5 (7.0)**
Anti-social	1 (1.4)
Emotionally instable	4 (5.6)
Unspecified	1 (1.4)
Mixed type	1 (1.4)
**Autism spectrum disorders (F8)**	**1 (1.4)**
Atypical autism	1 (1.4)
Asperger syndrome	1 (1.4)
**Attention deficit disorders (F9)**	**7 (9.9)**
Attention deficit disorder (ADD)	2 (2.8)
Attention deficit hyperactivity disorder (ADHD)	2 (2.8)
Unspecified	3 (4.2)
**Any psychiatric diagnose**	**51 (71.8)**

* Other than opioids, as opioid dependence is a criterion for study-inclusion. ** F1–F9 refers to the ICD-10 classification used in the medical journals. Substance abuse is here defined for those with abuse only. If patients had both an abuse and dependence diagnosis of one and the same substance, only the latter is counted.
